# Coronary microvascular dysfunction in patients with stable coronary artery disease: The CE-MARC 2 coronary physiology sub-study

**DOI:** 10.1016/j.ijcard.2018.04.061

**Published:** 2018-09-01

**Authors:** David Corcoran, Robin Young, David Adlam, Alex McConnachie, Kenneth Mangion, David Ripley, David Cairns, Julia Brown, Chiara Bucciarelli-Ducci, Andreas Baumbach, Rajesh Kharbanda, Keith G. Oldroyd, Gerry P. McCann, John P. Greenwood, Colin Berry

**Affiliations:** a British Heart Foundation (BHF) Glasgow Cardiovascular Research Centre, University of Glasgow, Glasgow, Scotland, UK; b West of Scotland Heart and Lung Centre, Golden Jubilee National Hospital, Glasgow, Scotland, UK; c Robertson Centre for Biostatistics, University of Glasgow, Scotland, UK; d Department of Cardiovascular Sciences, University of Leicester and the NIHR Leicester Biomedical Research Centre, Leicester, UK; e Leeds Institute of Cardiovascular and Metabolic Medicine, University of Leeds, Leeds, UK; f Leeds Institute of Clinical Trials Research, University of Leeds, Leeds, UK; g Bristol Heart Institute, University of Bristol, Bristol, UK; h Oxford Heart Centre, John Radcliffe Hospital, Oxford, UK

**Keywords:** Coronary artery disease, Coronary microvascular dysfunction, Angina, Stable ischaemic heart disease, CMR, cardiovascular magnetic resonance, CE-MARC 2, Clinical Evaluation of Magnetic Resonance Imaging in Coronary Heart Disease 2, CAD, coronary artery disease, CFR, coronary flow reserve, CTCA, computed tomography coronary angiography, FFR, fractional flow reserve, IMR, index of microcirculatory resistance, IHD, ischaemic heart disease, MPS, myocardial perfusion scintigraphy, NICE, National Institute for Health and Care Excellence, NOCAD, no obstructive coronary artery disease, RRR, resistance reserve ratio

## Abstract

**Background:**

In patients with angina undergoing invasive management, no obstructive coronary artery disease (NOCAD) is a common finding, and angina may persist following percutaneous coronary intervention (PCI). Coronary microvascular dysfunction may be relevant. We aimed to assess the proportion of patients presenting with suspected CAD who had coronary microvascular dysfunction.

**Methods:**

Clinical Evaluation of Magnetic Resonance Imaging in Coronary Heart Disease 2 (CE-MARC 2) was a prospective multicenter randomised controlled trial of functional imaging versus guideline-based management in patients with suspected CAD. Invasive coronary angiography was protocol-directed. Fractional flow reserve (FFR) and parameters of microvascular function (coronary flow reserve (CFR), index of microcirculatory resistance (IMR), resistance reserve ratio (RRR)) were measured in major epicardial coronary arteries with ≥40–≤90% diameter stenosis. An FFR value ≤0.80 indicated the presence of obstructive CAD.

**Results:**

267/1202 (22.2%) patients underwent angiography and 81 (30%) patients had FFR measured. 63 (78%) of these patients had microvascular function assessed in 85 arteries (mean age 58.5 ± 8.2 years; 47 (75%) male). 25/63 (40%) patients had NOCAD, and of these, 17 (68%) had an abnormality ≥1 parameter of microvascular function (abnormal IMR (≥25), abnormal CFR (<2.0), and abnormal RRR (<2.0) occurred in 10 (40%), 12 (48%), and 11 (44%), respectively). 38/63 (60%) patients had obstructive epicardial CAD. Of these patients, 15/38 (39%), 20/38 (53%), and 12/38 (32%) had an abnormal IMR, CFR and RRR, respectively.

**Conclusions:**

Coronary microvascular dysfunction is common in patients with angina. Invasive assessment of microvascular function may be informative and relevant for decision-making in patients with both NOCAD and obstructive epicardial CAD.

**Clinical trial registration:**

ClinicalTrials.gov Identifier: NCT01664858

## Highlights

• In patients with angina undergoing invasive diagnostic management, no obstructive coronary artery disease (NOCAD) is a common finding.
• In a contemporary, multicenter, prospectively enrolled population of patients presenting with stable angina, 63 patients underwent invasive measurement of microvascular function and 45 (71%) of these had evidence of coronary microvascular dysfunction. Twenty-five (40%) patients had NOCAD and 17 (68%) of these patients had microvascular dysfunction.
• Our results demonstrate that microvascular dysfunction is highly prevalent in patients with stable angina. In patients with NOCAD, ischaemia due to microvascular dysfunction may provide an explanation for chest pain symptoms.
• Further work is required to determine whether routine invasive measurement of coronary microvascular function leads to improved diagnosis, targeted therapeutics, and long-term health outcomes.

## 1 Introduction

Ischaemic heart disease (IHD) persists as the leading global cause of death standardised by age and sex [1]. A considerable proportion of patients with angina have non-flow limiting (non-obstructive) epicardial coronary artery disease (CAD) [2]. Angina may persist in patients following technically successful percutaneous coronary intervention (PCI) as reported by the Percutaneous Coronary Intervention in Stable Angina (ORBITA) trial investigators [3]. Although several factors may be relevant, ‘microvascular angina’ is an explanation in some patients [[4], [5], [6]]. Microvascular angina is prognostically important [[7], [8], [9]] and clinical management is supported by contemporary practice guidelines [10]. Given the challenges in diagnosing and treating microvascular angina in clinical practice, it is a condition of unmet clinical need [11].

Advances in interventional diagnostic techniques [12,13] enable new insights into coronary microvascular function in patients with stable CAD [14]. Invasive diagnostic tests identify abnormalities in vasodilator capacity (coronary flow reserve, CFR; resistance reserve ratio, RRR) and microvascular resistance (index of microcirculatory resistance, IMR) in patients with obstructive or no obstructive epicardial CAD (NOCAD). The availability of these invasive tests provides a reference dataset for comparison against non-invasive functional testing.

We aimed to assess the prevalence of coronary microvascular dysfunction in patients with suspected stable IHD who had been referred for invasive coronary angiography in the Clinical Evaluation of Magnetic Resonance Imaging in Coronary Heart Disease 2 (CE-MARC 2) clinical trial (ClinicalTrials.gov Identifier: NCT01664858) [15]. CE-MARC 2 was a UK prospective multicentre three-arm parallel group, randomised controlled trial of routine functional imaging versus guideline-based management in patients presenting with suspected angina. In this pre-specified sub-study, the primary aim was to assess the proportion of patients with abnormal coronary microvascular function, in those with both NOCAD and obstructive epicardial CAD. Secondary aims were to assess the associations between clinical characteristics and abnormal coronary microvascular function, and between non-invasive diagnostic tests for stable IHD and abnormalities in coronary microvascular function.

## 2 Methods

### 2.1 Study population

CE-MARC 2 randomised 1202 patients (2:2:1) to 3.0 Tesla stress perfusion cardiovascular magnetic resonance (CMR), myocardial perfusion scintigraphy (MPS) (according to American College of Cardiology/American Heart Association appropriate-use criteria), or to management based on the pre-test probability of CAD (10–29%: CT (computed tomography) calcium scoring ± CTCA (CT coronary angiography); 30–60%: MPS; 61–90%: invasive coronary angiography) [16]. The UK National Institute for Health and Care Excellence (NICE) clinical practice guidelines recommend non-invasive functional testing for patients for the assessment of angina in patients with confirmed CAD [17]. Patients age ≥30 years with known or suspected angina, a pre-test likelihood of epicardial CAD 10–90%, and who were deemed suitable for myocardial revascularisation were enrolled. Key exclusion criteria included non-anginal chest pain, a normal MPS or CTCA result <2 years previously, prior myocardial infarction, prior coronary revascularisation, and contraindication to MPS, CMR, or CTCA. Following enrolment, patients were referred for invasive coronary angiography following either i) positive CMR, MPS, or CTCA, ii) patients with a pre-test probability of CAD 61–90% randomised into the NICE-guided strategy, iii) inconclusive CMR, MPS, or CTCA, iv) clinician decision.

Following invasive angiography, coronary arteries ≥2.5 mm with a visually-assessed ≥40–≤90% diameter stenosis underwent protocol-directed fractional flow reserve (FFR) measurement to assess for flow-limiting epicardial CAD. The invasive coronary physiology recordings were prospectively collected from the 6 study sites for analysis in the Glasgow Coronary Physiology Core Laboratory. Recordings were analysed off-line using RADIVIEW™ (St. Jude Medical, St. Paul, MN) by 2 experienced observers (D.C. and C.B.). The Gensini score was calculated to quantify the epicardial CAD burden [18].

### 2.2 Coronary physiology protocol

Coronary physiology measurements were performed with a dual coronary pressure- and temperature-sensitive guide wire (Certus™, St. Jude Medical, St. Paul, MN). Arteries undergoing FFR measurement also had parameters of microvascular function assessed. The coronary guidewire was calibrated outside the body, equalised with aortic pressure at the coronary guide catheter ostium, and advanced to the distal third of the epicardial coronary artery undergoing interrogation. Resting thermodilution was performed using 3 intracoronary boluses of room temperature 0.9% saline. Maximal hyperaemia was then induced by an intracoronary bolus of 200 μg nitrate and a 3-min peripheral intravenous adenosine infusion (140 μg/kg/min), and hyperaemic thermodilution was performed using 3 intracoronary boluses of room temperature 0.9% saline. Mean aortic and distal coronary pressures were recorded during rest and maximal hyperaemia, and the mean resting and hyperaemic transit times derived.

The invasive measurements of epicardial and microvascular function were: 1. FFR describes the functional significance of an epicardial stenosis: FFR = distal coronary pressure (P_d_)/aortic pressure (P_a_), at maximal hyperaemia [19]; 2. CFR describes the vasodilatory capacity of the epicardial and microvascular coronary compartments: CFR = mean resting transit time/mean hyperaemic transit time [20]; 3. IMR is a measure of coronary microvascular resistance: IMR = P_d_ × mean transit time, at maximal hyperaemia [21]. IMR was uncorrected for coronary wedge pressure [22]; 4. Resistance reserve ratio (RRR) is a measure of the vasodilatory capacity of the microcirculation: RRR = RI/IMR, (RI = baseline resistance index, calculated as: resting P_d_ × mean resting transit time [23].

### 2.3 Non-invasive ischaemia imaging

The CMR and MPS protocols used in the CE-MARC 2 trial have been previously described [15]. Non-invasive ischaemia tests that were reported as demonstrating ‘significant ischaemia’ or ‘inconclusive’ triggered referral for invasive coronary angiography. Significant ischaemia was defined as: i) CMR criteria: any segment with transmural ischaemia, ischaemia >60° (basal/mid) or >90° (apical) of left ventricular circumference, or ischaemia in ≥2 adjacent segments; ii) MPS criteria: summed stress score ≥4 in patients.

### 2.4 Definitions of obstructive epicardial coronary artery disease and microvascular dysfunction

The cohort was dichotomised into obstructive CAD and NOCAD on a per-patient and per-vessel basis. For the per-patient analysis, obstructive CAD was defined as any epicardial coronary stenosis with FFR ≤0.80, or if FFR was not measured in an artery, a diameter stenosis of ≥70% in 1 angiographic view or ≥50% or higher in 2 orthogonal views assessed by quantitative coronary angiography (QCA), as per the CE-MARC2 protocol [24]. For the per-vessel analysis, an FFR threshold of ≤0.80 defined an artery with obstructive epicardial CAD. Microvascular dysfunction was defined as an abnormality in one or more parameters that reflect distinct properties of the microcirculation: increased microvascular resistance (IMR ≥25)), impaired coronary vasodilator reserve (CFR <2.0), and impaired microvascular vasodilator capacity (RRR <2.0) [7,13,23].

### 2.5 Statistical analyses

The primary outcome was the prevalence of coronary microvascular dysfunction in patients undergoing invasive management. The prevalence of microvascular dysfunction in participants with NOCAD and obstructive epicardial CAD were prioritised secondary outcomes. Results are expressed as mean ± SD (range). Fisher's tests were used to compare categorical variables and one-way ANOVA was used to compare continuous variables between patients with NOCAD and obstructive epicardial CAD. Variables associated with epicardial CAD were tested for their ability to predict microvascular dysfunction in univariate binary logistic regression analyses. Linear regression analysis was performed to assess the relationship between parameters of microvascular function. A two-sided value of p < 0.05 was considered significant. Statistical analyses were performed with SPSS 15.0 (SPSS, Chicago, IL).

### 2.6 Sample size considerations

Pilot data indicated that FFR would be measured in approximately 10% (n = 120) of the CE-MARC 2 participants undergoing invasive coronary angiography. This proportion of participants undergoing FFR measurement and protocol-directed microvascular function testing was anticipated to give clinically-meaningful results on the frequency of microvascular dysfunction in symptomatic patients with angina, including those with NOCAD and obstructive CAD.

## 3 Results

### 3.1 Characteristics of the participants undergoing invasive management

**Fig. 1:**
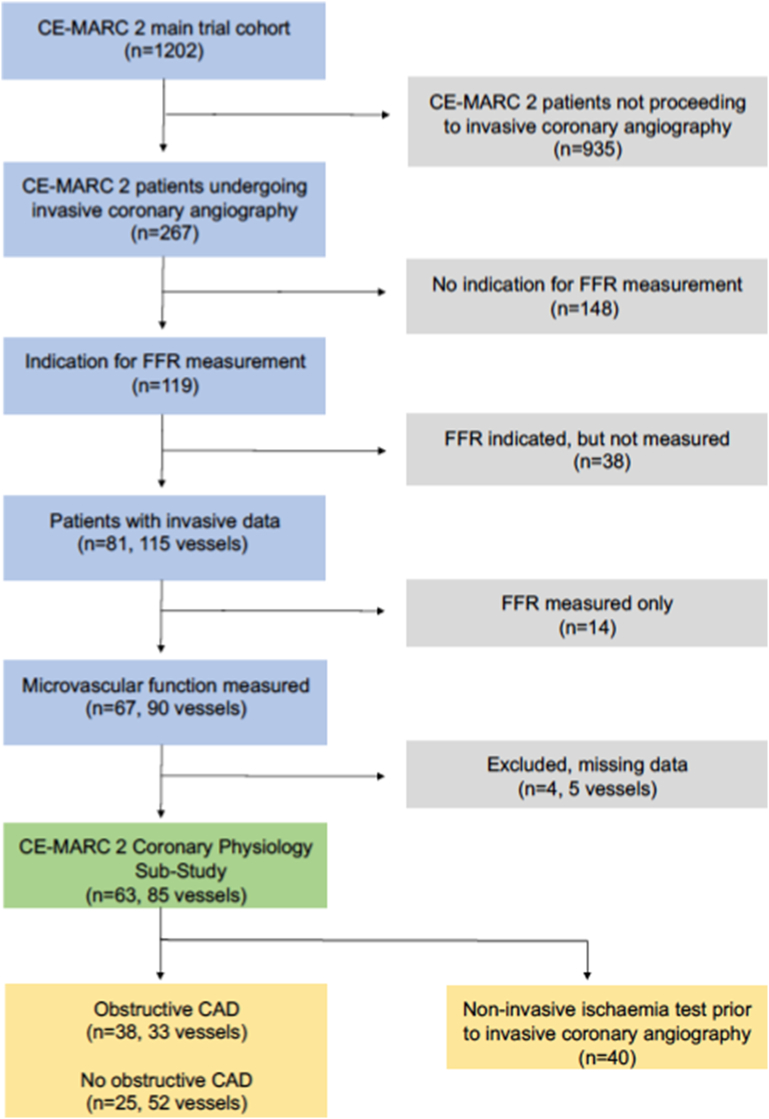
CONSORT flow diagram. CONSORT flow diagram of patients enrolled in the CE-MARC 2 microvascular sub-study. Of 1202 patients enrolled into the CE-MARC 2 trial, 267 (22.2%) underwent invasive coronary angiography and 119 (45%) of these patients had an indication for FFR measurement. Thirty-eight (32%) of these patients did not have FFR measured for the following reasons: severe obstructive CAD, n = 15; urgent invasive management, n = 3; clinical and technical factors, n = 20. Eighty-one patients had FFR measured in at least one coronary artery (115 vessels) and additional parameters of microvascular function were available for 63/81 (78%) patients in 85 vessels. Overall, 63 patients had coronary microvascular function assessed, including 22, 18 and 23 patients randomised to CMR-guided, MPS-guided and NICE management-guided care, respectively. FFR = fractional flow reserve.

The flow of participants referred for invasive assessments in the trial is shown in Fig. 1. Eighty-one patients had FFR measured in at least one coronary artery (115 vessels). Parameters of microvascular function were available in 85 coronary arteries from 63/81 (78%) patients. Logistical reasons limited microvascular measurements in the remaining 18 patients.

The study population (n = 63) included 22, 18 and 23 patients who had been randomised to CMR-guided, MPS-guided and NICE guideline-based care, respectively. Their baseline clinical characteristics are shown in Table 1. All patients had stable anginal symptoms, with 30 patients classified as having typical symptoms and 33 having atypical symptoms.

**Table 1:** Baseline clinical characteristics of the study participants.

Variable	n = 63
Age, years	58.5 ± 8.2
Male sex, n (%)	47 (75)
Height, m	1.71 ± 0.10
Weight, kg	83.4 ± 14.0
BMI, kg/m^2^	28.3 ± 3.8
Creatinine, μmol/l	79.4 ± 20.5
Gensini score	11.6 ± 10.3
*Medical history: n (%)*	
Chest Pain	
Typical	30 (48)
Atypical	33 (52)
Hypertension	32 (51)
Diabetes mellitus	10 (16)
Hypercholesterolemia	27 (43)
Current smoker	14 (22)
Ex-smoker	23 (37)
Never smoker	26 (41)
Family history of CAD	36 (57)
Cerebrovascular disease	1 (2)
Peripheral vascular disease	1 (2)
*Baseline medical therapy: n (%)*	
Anti-platelet	46 (73)
Angiotensin converting enzyme inhibitor	17 (27)
Angiotensin receptor blocker	8 (13)
Statin	34 (54)
ß-blocker	37 (59)
Calcium channel blocker	12 (19)
Oral nitrate	6 (10)
Nicorandil	1 (2)
Oral hypo-glycemic agent	6 (10)
Insulin	2 (3)

Variables are presented as mean ± SD or n (%) as appropriate. BMI = body mass index, GTN = glyceryl trinitrate, CAD = coronary artery disease.

### 3.2 Per-patient invasive coronary physiology analysis

#### 3.2.1 Coronary physiology tests

**Fig. 2:**
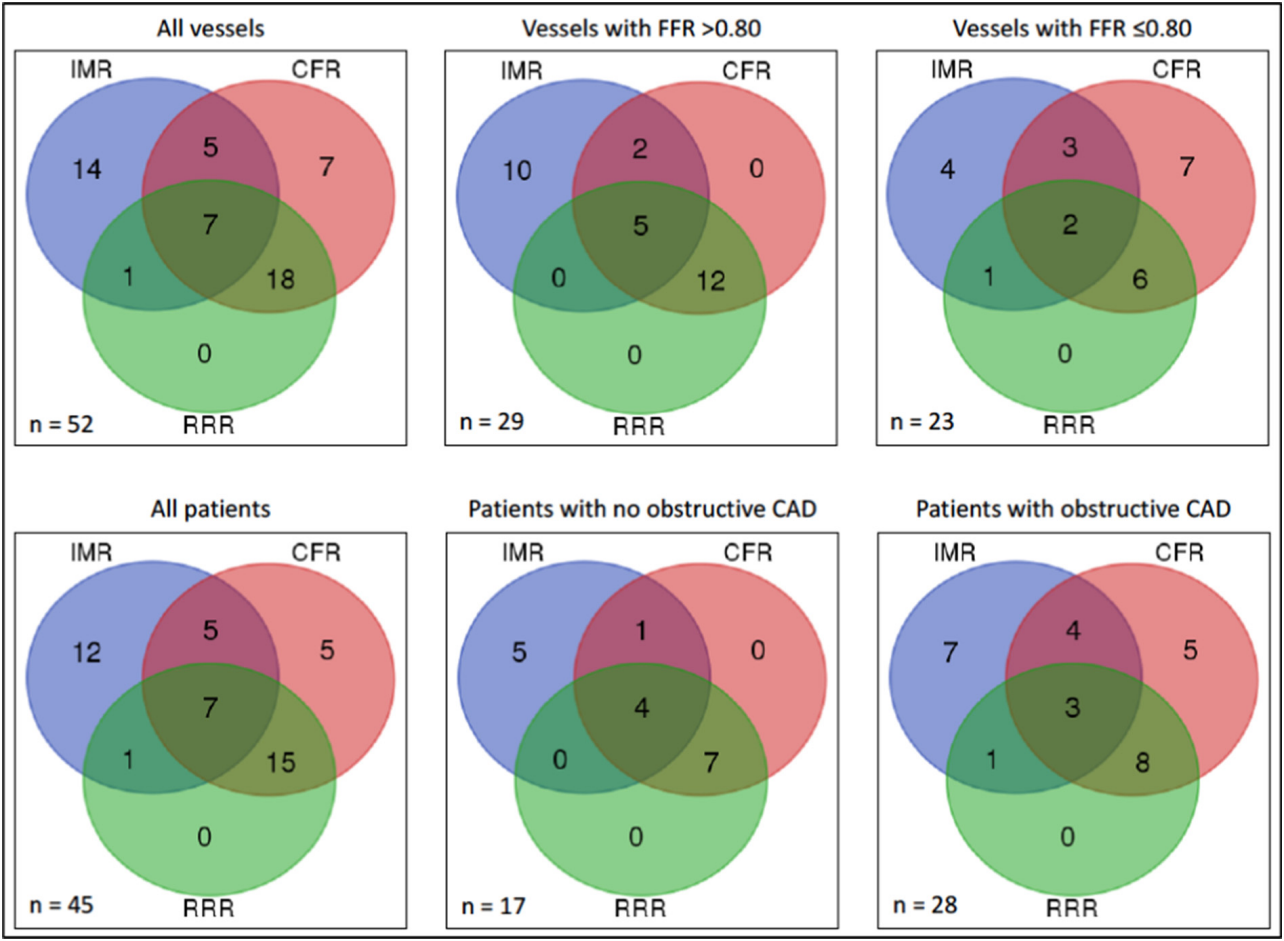
Abnormal invasive coronary microvascular function test results. Abnormal IMR, CFR and RRR on a per-patient and per-vessel basis. FFR = fractional flow reserve, IMR = index of microcirculatory resistance, CFR = coronary flow reserve, RRR = resistance reserve ratio.

Forty-five (71%) of the 63 patients had at least one abnormal parameter reflecting coronary microvascular dysfunction (Fig. 2). Patients with more than one vessel undergoing microvascular function measurement (n = 20, 32%) had the artery with the most abnormal microvascular function included in the analysis.

Twenty-five (40%) patients were classified as having NOCAD. Of these patients, 17/25 (68%) had an abnormality in at least one measurement of coronary microvascular function, and 5 (20%) patients had concordantly abnormal IMR and CFR values.

Thirty-eight patients (60%) were classified as having obstructive epicardial CAD. Of these patients, 15/38 (39%) had an abnormal IMR, and 12/38 (32%) had an abnormal RRR. A reduced CFR concordant with the abnormal FFR, was present in 20/38 (53%) patients.

#### 3.2.2 Comparison of baseline clinical characteristics

The clinical characteristics of patients with obstructive and NOCAD were similar, and the parameters of microvascular function were also similar between the groups (Table 2).

**Table 2:** Comparison of baseline characteristics and coronary physiology in patients with NOCAD and obstructive epicardial CAD.

Variable	NOCAD (n = 25)	Obstructive CAD (n = 38)	*p* value
*Baseline characteristics*			
Age, years	59.0 ± 8.5	58.2 ± 8.2	0.71
Male sex	16 (64%)	31 (82%)	0.15
Hypertension	13 (52%)	19 (50%)	0.99
Diabetes mellitus	4 (20%)	5 (13%)	0.50
Smoking history:			0.44
Current smoker	7 (28%)	7 (18%)	
Ex-smoker	10 (40%)	13 (34%)	
Never smoker	8 (32%)	18 (47%)	
Hypercholesterolemia	11 (41%)	19 (48%)	0.63
Gensini score	13.2 ± 11.8	10.4 ± 9.1	0.31
*Invasive coronary physiology results*			
FFR	0.91 ± 0.06	0.75 ± 0.14	<0.0001
IMR	25.6 ± 14.0	22.9 ± 13.1	0.44
RRR	2.4 ± 0.8	2.7 ± 1.2	0.34
CFR	2.2 ± 0.9	2.0 ± 0.9	0.56
Baseline resistance index	63.0 ± 49.6	61.0 ± 47.4	0.88

Variables are mean ± SD and n (%) where appropriate. FFR = fractional flow reserve, IMR = index of microcirculatory resistance, CFR = coronary flow reserve, RRR = resistance reserve ratio, RI = baseline resistance index. CAD = coronary artery disease.

In a univariate binary logistic regression analysis of variables associated with obstructive epicardial CAD, females were more likely to exhibit an abnormal IMR than males (OR 3.56 95% CI 1.09–11.61, p = 0.04). There were no statistically significant associates of abnormal CFR or abnormal RRR. There was no statistically significant difference in the proportion of patients with abnormal microvascular dysfunction who underwent direct invasive coronary angiography (pre-test probability of CAD 61–90% randomised into the NICE guideline-based care) compared with those who had a prior non-invasive ischaemia test (abnormal CFR p = 1.00, abnormal IMR p = 0.79, abnormal RRR p = 0.60). There was no statistically significant difference in the proportion of patients with typical or atypical chest pain symptoms and either abnormal CFR or abnormal IMR (p = 0.45 and p = 1.00, respectively). In patients with atypical chest pain symptoms, there was a statistically significant difference in the proportion with an abnormal compared to normal RRR (26 vs. 7 patients, p = 0.10).

#### 3.2.3 Non-invasive ischaemia testing

Forty of the 63 patients had a non-invasive ischaemia test performed prior to invasive angiography (comprised of patients randomised to CMR-guided (n = 22), and MPS-guided (n = 18) care). Of these 40 patients, 31 (78%) demonstrated significant ischaemia, and 9 (22%) patients had an inconclusive study. Fourteen (35%) patients had NOCAD, of whom 9 (64%) had significant ischaemia and 5 (36%) an inconclusive study. In total 10/14 (71%) patients with NOCAD had abnormal invasive coronary microvascular function tests. There were no differences in the non-invasive ischaemia test results (either significant ischaemia or inconclusive study) and the number of patients with microvascular dysfunction.

### 3.3 Per-vessel invasive coronary physiology analysis

Microvascular function was assessed in 85 coronary arteries (left anterior descending artery n = 51 (60%), circumflex artery n = 12 (14%), right coronary artery n = 22 (26%)). 52/85 (61%) arteries had an abnormality in at least one measurement of microvascular function (Fig. 2).

Of the 85 arteries studied, 52 (61%) were classified as having NOCAD by FFR measurement. An abnormality in at least one measurement of microvascular function occurred in 29/52 (56%) arteries (abnormal IMR in 17/52 (33%), abnormal CFR in 19/52 (36%), and abnormal RRR in 17/52 (33%)). 7/52 (14%) arteries had concordantly abnormal IMR and CFR values.

In the 33/85 (39%) arteries with obstructive CAD, 23/33 (70%) arteries had an abnormality in at least one measurement of microvascular function microvascular dysfunction (abnormal IMR in 10/33 (30%) arteries, and abnormal RRR in 9/33 (27%) arteries. A reduced CFR concordant with the abnormal FFR, was present in 18/33 (55%) arteries, and 5/33 (15%) arteries had concordantly abnormal IMR and CFR values.

There was a weak, positive correlation between CFR and FFR (r = 0.28, R^2^ = 0.08, p = 0.01). There was no correlation between RRR and FFR (r = 0.06, R^2^ = 0.004, p = 0.56), or IMR and FFR (r = 0.03, R^2^ = 0.001, p = 0.81). There was a strong, positive correlation between RRR and CFR (r = 0.91, R^2^ = 0.83, p < 0.001).

## 4 Discussion

We have shown that in an invasively managed population of patients with stable symptoms prospectively enrolled in a contemporary, multicentre, clinical trial, coronary microvascular dysfunction was common. Specifically, 45 (71%) of 63 patients who had the relevant tests had invasive evidence of coronary microvascular dysfunction, and in patients with NOCAD a high proportion (17/25 (68%)) also had invasive evidence of microvascular dysfunction. Secondly, microvascular dysfunction was also a frequent finding in patients with obstructive epicardial CAD.

The distributions of CFR and IMR values indicate that these parameters reflect distinct properties of coronary microvascular function and are not interchangeable [12]. CFR reflects the vasodilator capacity of the epicardial coronary artery and its subtended microvasculature, whereas IMR is a direct measure of microvascular resistance. Our results implicate abnormalities in microvascular function as a potential explanation for abnormal false-positive non-invasive ischaemia test results in symptomatic patients without obstructive epicardial CAD. For example, 14 (56%) of 25 patients with NOCAD underwent a non-invasive ischaemia test prior to invasive coronary angiography, and 9/14 patients (64%) had evidence of clinically significant ischaemia. By protocol, such results would be classified as false-positive when considered against invasive findings of NOCAD (i.e. FFR >0.80), leading to false reassurance for patients and given the practice guideline recommendations for microvascular angina [10], potentially sub-optimal management. 71% of the patients with either positive or inconclusive non-invasive stress tests had abnormal IMR, CFR or RRR, when assessed invasively. This finding is consistent with prior literature, which describes subendocardial perfusion abnormalities in patients with microvascular dysfunction [25]. In contrast to qualitative assessment, fully quantitative stress perfusion CMR and positron emission tomography (PET) allow for quantification of absolute myocardial blood flow (in ml/g/min) and myocardial perfusion reserve (equivalent to CFR), and these tests may be used to assess microcirculatory function following exclusion of obstructive epicardial CAD [9,26,27]. An impairment in vasodilator capacity during stress (e.g. secondary to diffuse atherosclerosis) or increased microvascular resistance (e.g. secondary to microvascular rarefaction or remodeling) may cause a supply-demand mismatch in myocardial perfusion, leading to ischaemia and symptoms in the absence of obstructive CAD or other relevant systemic health problems (e.g. anaemia).

In CE-MARC 2, the primary endpoint was a protocol-defined unnecessary invasive coronary angiogram [15]. Of 139 patients with this primary endpoint, 39 (28%) had a false-positive or inconclusive non-invasive test (21 with CMR, 13 with MPS, and 5 undergoing non-invasive imaging in the NICE guideline-based care group) [15]. Our results indicate that if invasive parameters of microvascular function were considered, the rate of false-positive tests results in the CE-MARC 2 trial may have been lower, but rather than the selective approach in our study, a future protocol should involve a systematic measurement of coronary microvascular function in order to more broadly assess disease prevalence in symptomatic patients.

Our data are consistent with other contemporary natural history studies that demonstrate a high prevalence of microvascular dysfunction in patients with angina but angiographically unobstructed coronaries. Reis et al. in a sub-study of patients enrolled in the NHLBI-sponsored Women's Ischemia Syndrome Evaluation (WISE) study, assessed CFR with Doppler-derived flow velocity measurements in 159 females with chest pain and NOCAD on invasive angiography [28]. Seventy-four (47%) women had abnormal CFR (<2.5) consistent with microvascular dysfunction. Lee et al. performed a comprehensive invasive assessment of coronary function in 139 patients with angina and NOCAD, and found that the majority of patients had at least one abnormality to account for their symptoms (abnormal IMR (≥25) in 29 (21%) patients, and abnormal coronary endothelial function in response to acetylcholine testing in 61 (44%) patients) [13].

We found female sex to be the only predictor of abnormal microvascular function (IMR ≥25). Recent studies have reported conflicting associations between female sex and microvascular dysfunction. The International Index of Microcirculatory Resistance Registry measured IMR in 1096 patients (1452 vessels) undergoing elective invasive management. Female sex was a predictor of an elevated IMR, along with prior myocardial infarction, right coronary artery, and obesity [29]. Kobayashi et al. measured IMR and CFR in the left anterior descending artery of 147 patients with angiographically unobstructed coronaries. There was no sex difference in IMR, but CFR was lower in females than males predominantly due to shorter resting thermodilution transit times in females, and female sex was an independent predictor of reduced CFR [30]. However, Murthy et al. enrolled 1218 patients with suspected angina referred for stress positron emission tomography (PET) imaging. Patients were included if there was no evidence of obstructive epicardial CAD on PET imaging (assessed semi-quantitatively), and using a CFR threshold <2.0 microvascular dysfunction was prevalent in men and women (51% and 54% respectively) [9].

We found microvascular dysfunction to be common in patients with angina and obstructive epicardial CAD. The results implicate microvascular dysfunction as a potential explanation for recurrent angina following myocardial revascularisation [31,32]. In a meta-analysis of 5 randomised trials (n = 5286) comparing PCI and optimal medical therapy in patients with stable angina, 20.3% of patients undergoing PCI had persistent angina [33]. Similarly, the ORBITA trial randomised 230 patients with angina and single vessel CAD to PCI or a sham procedure [3]. There was no significant improvement in exercise time or angina with PCI beyond the effect of the sham procedure. A number of potential mechanisms for recurrent angina following revascularisation of obstructive epicardial CAD (i.e. Type 3 coronary microvascular dysfunction) have been proposed, including endothelial dysfunction [34], adverse microvascular remodeling distal to epicardial disease resulting in impaired microvascular vasodilator capacity and increased microvascular resistance [35], and anatomical and functional microvascular dysfunction due to distal embolisation of atherosclerotic material post-PCI [36]. Our results implicate microvascular dysfunction as being a relevant, potential explanation for the lack of benefit associated with PCI in some patients.

In patients with atypical chest pain symptoms, there was a statistically significant difference in the proportion with an abnormal compared to normal RRR (26 vs. 7 patients, p = 0.10). Given the small patient numbers, and that this association was only demonstrated with one parameter of microvascular function, the significance of this result is uncertain and merits further study.

Our results also provide new insights into the possibility of false-negative results with anatomical imaging methods, such as CTCA. In SCOT-HEART, 4146 patients referred for investigation of known or suspected angina were randomly assigned to standard care plus CTCA-guided management (n = 2073) or standard care (n = 2073) alone [37]. In a pre-specified analysis, symptoms and quality of life assessed at baseline, 6 weeks and 6 months, improved less in patients assigned to the CTCA-guided strategy as compared to standard care [38]. Whilst several factors may be relevant, one potential explanation is that some of the patients with NOCAD had microvascular or vasospastic angina leading to false reassurance and suboptimal management. The natural history of ischaemia in patients with NOCAD is being prospectively assessed in the CIAO-ISCHEMIA (NCT02347215) sub-study of the ISCHEMIA trial (NCT01471522). Our results may remind clinicians that measurement of coronary microvascular function may be helpful in patients with anginal symptoms and a negative invasive or non-invasive coronary angiogram. The clinical utility of routine measurement of coronary artery function in appropriately selected patients is currently being assessed in the Coronary Microvascular Angina (CorMicA) trial (NCT03193294).

We used a thermodilution technique for assessing microvascular function as this method may be performed using the same coronary guidewire as for guideline-directed FFR measurement. This technique is straightforward and transferable to routine clinical practice [10]. Measurement of FFR, IMR, CFR, and RRR with one guidewire enables the focused interrogation of epicardial and microvascular function. Doppler flow assessment also enables measurement of microvascular function (coronary flow velocity reserve (CFVR) and hyperaemic microvascular resistance (hMR)), however this technique is less transferable to real-world clinical practice.

### 4.1 Limitations

The prevalence of epicardial CAD in the CE-MARC 2 trial was lower than estimated, with 119 (9.9%) patients having an indication for FFR measurement. Only a sub-set (n = 67 (56%)) of the participants undergoing FFR measurement had microvascular function assessed, mainly because of logistical reasons (e.g. lack of clinician experience with microvascular function testing). The interpretation of the results is limited by the sample size for the sub-study as a proportion of the total CE-MARC 2 main trial cohort. Almost one third of the participants with a protocol-directed indication for FFR lacked this measurement, which in turn resulted in a lower proportion of participants with microvascular data than anticipated. Notwithstanding this point, our analysis includes the largest number of participants with paired invasive measures of microvascular function and non-invasive measures of ischaemia derived in a multicentre setting. The core laboratory approach and multicentre design mitigate against single centre effects e.g. positive reporting bias, which we believe enhances the validity of our findings. In the CE-MARC 2 trial, coronary angiography was invoked in patients with evidence of non-invasive ischaemia, or patients with a high pre-test probability of obstructive epicardial CAD in the NICE guideline-directed care group. Potentially, patients with microvascular or vasospastic angina may not have been referred for invasive angiography as non-invasive CTCA and ischaemia testing may lack sensitivity for coronary microvascular dysfunction.

The proportion of patients with abnormalities in coronary artery function may be under-represented in our analysis because although coronary microvascular resistance (IMR) and epicardial and microvascular vasodilatory capacity (CFR and RRR) were systematically measured, assessment of coronary endothelial function and vasospasm testing with acetylcholine was not part of the CE-MARC 2 protocol [39]. Potentially, had these tests been performed, the prevalence of clinically significant abnormalities of coronary artery function would have been even higher [6,40].

## 5 Conclusions

Coronary microvascular dysfunction is a common finding in invasively managed patients with angina in both those with NOCAD and obstructive epicardial CAD. Our findings provide a potential explanation for why PCI may not be beneficial, especially when performed in patients who may have non-flow limiting CAD [3]. These results may explain the occurrence of apparently false-positive non-invasive ischaemia test results in some patients. Invasive assessment of microvascular function may be informative and relevant for decision-making in patients with both NOCAD and obstructive epicardial CAD. Further research is required to assess the prevalence of coronary microvascular dysfunction in patients with stable IHD, and ultimately to determine whether routine measurement of microvascular function leads to improved clinical care.

## Sources of funding

This study was funded by the British Heart Foundation (Clinical Research Training Fellowship to D.C. [FS/14/15/30661]; Special Project Grant [SP/12/1/29062]). Additional support was received from the Leeds Teaching Hospital Charitable Foundation and the National Institute for Health Research, through the Local Clinical Research Networks and the Leeds Clinical Research Facility.

## Disclosures

D.A was supported by the Leicester NIHR Biomedical Research Centre, and received research funding from St. Jude Medical and Astra Zeneca. K.M. was supported by a Fellowship from the BHF (FS/15/54/31639). RK was supported by the NIHR Oxford Biomedical Research Centre (BRC). C.B.D. was supported by the NIHR Biomedical Research Centre at the University Hospitals Bristol NHS Foundation Trust and University of Bristol. G.P.M. was supported by a NIHR Post-Doctoral Fellowship and a Career Development Fellowship (PDF-2011-04-051). C.B. was supported by a Senior Fellowship from the Scottish Funding Council and a British Heart Foundation Centre of Research Excellence award (RE/13/5/30177). K.G.O. has acted as a consultant to Abbott and Volcano. C.B. is named on institutional research and consultancy agreements between the University of Glasgow and Abbot, Menarini, and Siemens Healthcare. These companies were not involved in this research or the manuscript. The views expressed in this publication are those of the authors and not necessarily those of the NHS, the National Institute for Health Research or the Department for Health. There are no other potential conflicts of interest.

## Appendix A Supplementary data

Supplementary material Image 1
