# Supplementary material for: Coronary microvascular dysfunction in patients with stable coronary artery disease: The CE-MARC 2 coronary physiology sub-study
Source: Int J Cardiol. 2018 Sep 1;266:7–14. doi: 10.1016/j.ijcard.2018.04.061 (PMC6008494; doi:10.1016/j.ijcard.2018.04.061)

# Coronary microvascular dysfunction in patients with stable coronary artery disease: The CE-MARC 2 coronary physiology sub-study

# Online-only Tables and Figures

# Figure legends

## Figure 3.

Title: Distribution of invasive parameters of microvascular function in vessels with NOCAD (FFR >0.80).

Caption: Vessels with NOCAD (FFR >0.80) classified into 4 groups based on the coronary flow reserve (CFR) and index of microcirculatory resistance (IMR) values: Group A: high IMR, low CFR; Group B: high IMR, high CFR; Group C: low IMR, low CFR; Group D: low IMR, high CFR. Y-axis reference lines = IMR value 25, X-axis reference line = CFR value 2.0.

## Figure 4.

Title: Distribution of all vessels according to FFR and CFR.

Caption: Linear regression line r=0.28, R^2^=0.07, p=0.01. Y-axis reference lines = CFR value 2.0, X-axis reference line = FFR value 0.8.

## Figure 5.

Title: Distribution of all vessels according to FFR and RRR.

Caption: Y-axis reference lines = RRR value 2.0, X-axis reference line = FFR value 0.8.

## Figure 6.

Title: Distribution of all vessels according to FFR and IMR.

Caption: Y-axis reference lines = IMR value 25, X-axis reference line = FFR value 0.8.

## Figure 7.

Title: Distribution of all vessels according to RRR and CFR.

Caption: Linear regression line r=0.91, R^2^=0.83, p<0.001. Y-axis reference lines = RRR value 2.0, X-axis reference line = CFR value 2.0.

## Figure 8.

Title: CE-MARC 2 coronary physiology sub-study case example.

Caption: Case example of a patient with microvascular dysfunction enrolled in the CE-MARC2 coronary physiology sub-study. A 51-year-old male presented with typical Canadian Cardiovascular Society class 2 angina. The patient was an ex-smoker, and had no other risk factors for coronary artery disease. Perfusion CMR with pharmacological stress induced by intravenous (IV) infusion of adenosine (140 μg/kg/min) (A) demonstrated an inducible circumferential subendocardial perfusion defect in the basal segments (red arrows). The patient proceeded to invasive diagnostic coronary angiography, which demonstrated no obstructive epicardial coronary stenosis. In the right coronary artery (B) there was a 40% stenosis in the distal vessel (red arrow). This stenosis was interrogated with fractional flow reserve measurement (FFR) with an identical stress protocol (IV adenosine, 140 μg/kg/min) (C). The FFR value was 0.99 consistent with a non-flow limiting epicardial stenosis. Microvascular function testing demonstrated a preserved coronary flow reserve (CFR) value of 3.0. The index of microcirculatory resistance (IMR) was markedly elevated at 54. The variation in the profiles of the thermodilution curves is less pronounced during hyperaemia than under resting conditions. The patient was diagnosed with coronary microvascular dysfunction (Type 1) and commenced on appropriate secondary prevention and anti-anginal therapy.

# Online-only Tables

**Table 3.** Predictors of abnormal coronary microvascular function.

| **Binary logistic regression** | **OR (CI)** | **P value** |
| --- | --- | --- |
| **Abnormal IMR (≥25)** | | |
| Female sex | 3.56 (1.09-11.61) | 0.04 |
| Hypercholesterolemia | 0.41 (0.15-1.15) | 0.09 |
| Age, years | 1.04 (0.97-1.10) | 0.22 |
| Smoking history:  - Ex-smoker  - Current smoker | 0.62 (0.20-2.00)  0.65 (0.17-2.50) | 0.42  0.65 |
| Hypertension | 1.20 (0.44-3.31) | 0.72 |
| Diabetes mellitus | 0.98 (0.25-3.91) | 0.98 |
| **Abnormal CFR (<2.0)** | | |
| None significant | | |
| **Abnormal RRR (<2.0)** | | |
| None significant | | |

In the analysis of smoking history, never smoker was the reference group. None of the variables were predictive of abnormal CFR (Female sex OR 1.90, p=0.28; Hypercholesterolemia OR 0.55, p=0.25; Age OR 1.05, p=0.17; Smoking history: Ex-smoker OR 1.27, p=0.67, Current smoker OR 1.56, p=0.51; Hypertension OR 0.64, p=0.38; Diabetes mellitus OR 0.64, p=0.53) or abnormal RRR (Female sex OR 1.06, p=0.92; Hypercholesterolemia OR 0.73, p=0.55; Age OR 0.99, p=0.80; Smoking history: Ex-smoker OR 0.70, p=0.56, Current smoker OR 1.20, p=0.79; Hypertension OR 0.92, p=0.87; Diabetes mellitus OR 1.41, p=0.64). IMR = index of microcirculatory resistance, CFR = coronary flow reserve, RRR = resistance reserve ratio, CAD = coronary artery disease.

**Table 4.** Summary of invasive and non-invasive abnormalities in patients with NOCAD.

| **Patients with NOCAD (n=25)** | | | | |
| --- | --- | --- | --- | --- |
| **All invasive microvascular function tests** | **IMR** | **CFR** | **RRR** | **Non-invasive ischaemia test results (n=14)** |
| Any abnormal  n=17 | ≥25  n=10 (40%) | <2.0  n=12 (48%) | <2.0  n=11 (44%) | Significant ischaemia n=9 (64%) |
| All normal  n=8 | <25  n=15 (60%) | ≥2.0  n=13 (52%) | ≥2.0  n=14 (56%) | Inconclusive result  n=5 (36%) |

IMR = index of microcirculatory resistance, CFR = coronary flow reserve, RRR = resistance reserve ratio, CAD = coronary artery disease.

**Table 5.** Per-vessel invasive coronary physiology results.

| **Variable** | **All vessels**  **(n=85)** | **No obstructive CAD**  **(n=52)** | **Obstructive CAD**  **(n=33)** |
| --- | --- | --- | --- |
| Resting mean transit time, s | 0.74±0.50 (0.14-2.55) | 0.74±0.51 (0.14-2.55) | 0.75±0.49 (0.16-2.45) |
| Hyperemic mean transit time, s | 0.34±0.26 (0.11-2.04) | 0.30±0.16 (0.12-0.81) | 0.41±0.35 (0.11-2.04) |
| Resting P_d_/P_a_ | 0.90±0.11 (0.36-1.00) | 0.96±0.04 (0.88-1.00) | 0.83±0.14 (0.36-0.95) |
| Fractional flow reserve | 0.81±0.14 (0.29-1.00) | 0.89±0.05 (0.81-1.00) | 0.68±0.13 (0.29-0.80) |
| IMR | 22.2±12.3 (4.4-67.0) | 22.6±11.4 (8.0-56.8) | 21.5±13.8 (4.4-67.0) |
| CFR | 2.3±1.1 (0.7-7.3) | 2.5±1.2 (1.0-7.3) | 2.0±0.85 (0.73-3.57) |
| RRR | 2.9±1.4 (1.1-9.9) | 2.9±1.5 (1.3-9.9) | 2.8±1.2 (1.1-5.0) |
| Basal resistance index | 61.5±43.9 (9.9-252.4) | 64.4±43.6 (14.1-232.1) | 57.0±44.7 (9.9-252.3) |
| Visual diameter stenosis severity, % | 58.9±15.5 (40-90) | 53.1±11.8 (40-90) | 68.0±16.4 (50-90) |
| *Coronary artery:*  - Left anterior descending artery  - Circumflex artery  - Right coronary artery | 51 (60%)  12 (14%)  22 (26%) | 28 (54%)  9 (17%)  15 (29%) | 23 (70%)  3 (9%)  7 (21%) |

Variables are mean±SD (range). CAD = coronary artery disease, IMR = index of microcirculatory resistance, CFR = coronary flow reserve, RRR = resistance reserve ratio.

# Online-only Figures

## Figure 3.


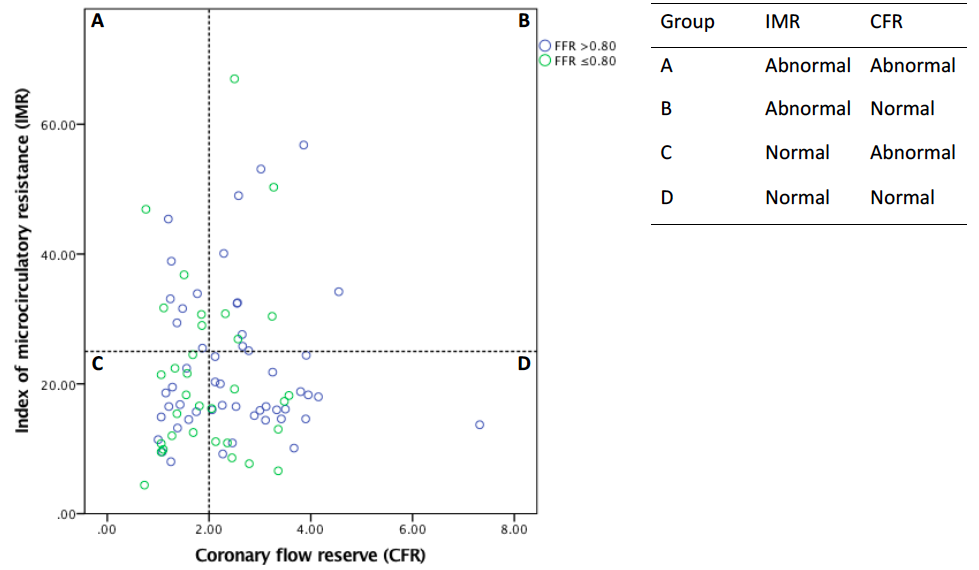


## Figure 4.


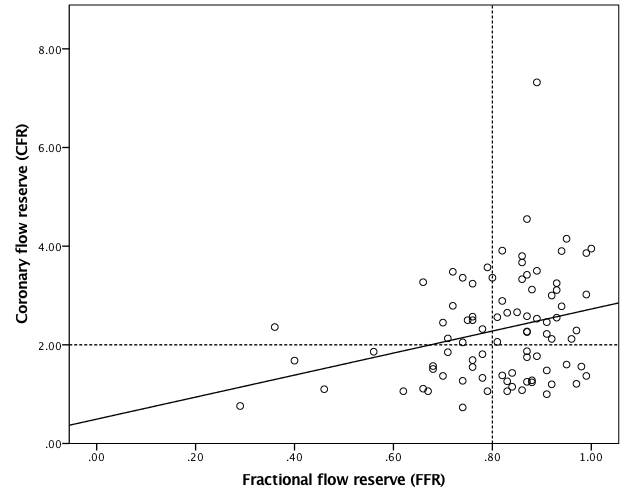


**Figure 5.**


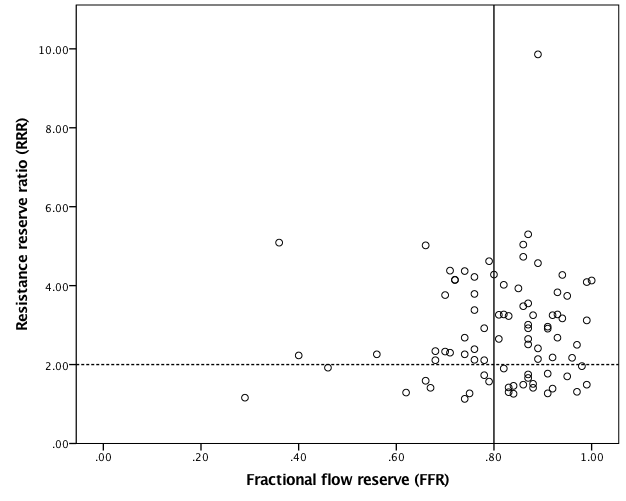


**Figure 6.**


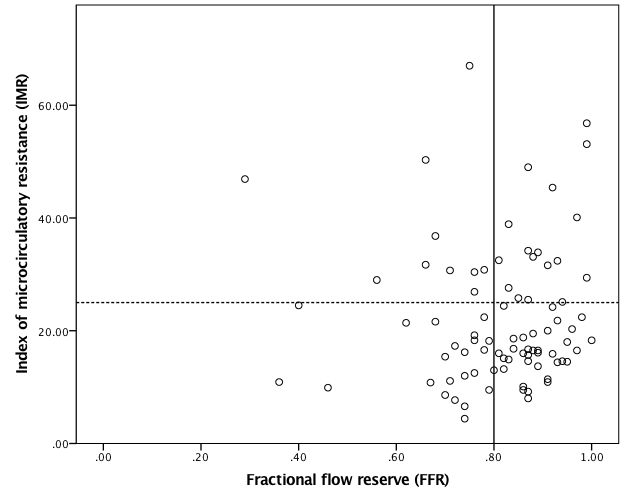


**Figure 7.**


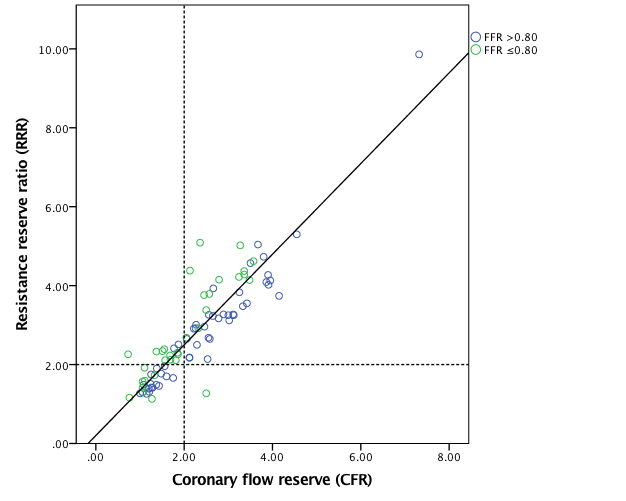


**Figure 8.**


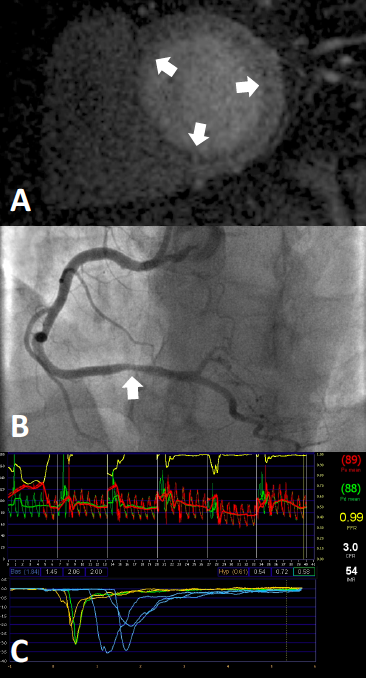

Supplement: Supplementary file 1 — Supplementary material [file mmc1.docx]
